# Improving Patient Satisfaction in the Hispanic American Community

**DOI:** 10.7759/cureus.27739

**Published:** 2022-08-07

**Authors:** Michael Kelson, Andrew Nguyen, Asaad Chaudhry, Patrick Roth

**Affiliations:** 1 Psychiatry, Hackensack Meridian School of Medicine, Nutley, USA; 2 Radiology, Hackensack Meridian School of Medicine, Nutley, USA; 3 Neurosurgery, Hackensack University Medical Center, Hackensack, USA

**Keywords:** limited english proficiency, spanish elective, medical students, medical school, patient satisfaction, population health, interpersonal and communication skills, language barrier

## Abstract

Hispanic Americans are the fastest growing ethnic group in the United States, with an ever-growing gap in the communicative capacity between patients and healthcare providers. This leads to linguistic marginalization and worse healthcare outcomes. There is an increasing need for Spanish literacy in healthcare professionals, including medical students. However, approximately half of medical schools don’t offer a Spanish elective. We performed a scoping review of the literature to assess the relationship between medical Spanish electives, verbal fluency, auditory comprehension, and student comfort. This study was conducted using PubMed and Google Scholar to evaluate articles on Spanish electives in medical schools. Nine articles met inclusion criteria. Almost all studies demonstrated benefit as per outcome measures assessed with statistical significance. The available literature supports the need for Spanish elective courses, with numerous advantages conferred, e.g. increased self-perceived knowledge about specific health issues in the Hispanic American community and reduction in inadvertent communication errors in the patient-provider-interpreter interaction. However, most of the reports analyzed exhibited numerous limitations that warrant future research studies in order to eliminate variables such as bias and issues with generalizability. The authors suggest that more medical schools offer virtual Spanish electives with a focus on empathetic language strategies and patient satisfaction.

## Introduction and background

Hispanic Americans are projected to comprise nearly 25% of the United States (US) population by 2050 [1,2]. Prior studies suggest that this ethnic group is the fastest growing subgroup in the United States, with a projected growth of 273% between 2000 and 2050 [3,4]. According to the US Census Bureau, the population of Latinos living in the US today is estimated at 62 million individuals [1]. Just in the last decade, we saw increases of 23%, which is reported to be 17% higher than the national average. Yet, prior studies report that only 6% of physicians identify as bilingual, with many Spanish-speaking patients having limited English proficiency [5,6]. Thus, communication barriers are becoming increasingly prevalent in this nation.

Language (the way in which one communicates) and health literacy (the degree to which individuals have the capacity to obtain, process, and understand basic health information needed to make appropriate decisions) are two vital components making up the social determinants of health, which directly influences health outcomes [7-9]. As Spanish is the fastest growing non-English language in the US, there is an increased need for bilingual physicians and healthcare professionals [3,4]. Based upon research we have on language concordance in the health services, we have seen that a shared language affects healthcare interactions on macro-, meso-, and micro-levels [10-12]. When speaking the same language as another individual, healthcare providers tend to ask more in-depth questions and spend a greater quality of time with patients [13,14]. From patients’ perspective, it has been shown that they display greater trust with their language concordant providers and are able to better understand and follow a physician’s medical advice or recommendations [14]. According to a cross-sectional study reviewing language barriers, physician-patient language concordance, and glycemic control among insured Latinos with diabetes, better health outcomes were positively correlated with physicians speaking to patients in their native language [15]. As it is termed, linguistic marginalization is a prevalent barrier that is recognized in healthcare today [7,9]. Despite an extensive body of evidence on the increased incidence of language barriers seen in clinical practice, there is an ever-growing gap in the communicative capacity between patients and healthcare providers [13,16]. One way to tackle this issue is through increased Spanish literacy in medical school students.

In this study, we performed a scoping review to evaluate the current research on medical Spanish electives offered. Several studies have assessed the effectiveness of these courses with outcome measures such as verbal fluency and listening skills [17-21]. We propose solutions to increase the prevalence and efficacy of medical Spanish electives offered to students during their fourth year of clinical instruction.

## Review

Methods

This study was conducted using PubMed and Google Scholar, for the period of January 2000 to July 2022. No restrictions were applied as per language or publication status. Keywords used include “language barriers,” “population health,” “patient satisfaction,” “medical school,” “Spanish elective” and “limited English proficiency” and Boolean operators AND/OR. We reviewed the literature pertaining to Spanish electives in medical schools and their efficacy in the setting of population health. This study compared data that addressed the impact of these courses on language retention and fluency in medical school students. We excluded studies that were not conducted in a medical school context, those that did not include medical school students, and studies that did not mention language barriers in the setting of communication difficulties. In total, 81 articles were found, of which nine were included in this review. Each article was reviewed by at least two authors for eligibility of inclusion, and all data was grouped into Excel spreadsheets (Microsoft, Redmond).

Results

The results from three studies identified assessed medical school opportunities to partake in medical Spanish courses [22-24] (Table 1).

**Table 1:** Summary of medical school programs offering Spanish curricula

Author	Objective or Question	Population and Design	Measured Outcomes	Main Findings
Maben et al. [22]	What options exist for medical students to learn Spanish?	Literature review of data from Medline, Association of American Medical Colleges, 125 school websites	Proportion of schools offering Spanish electives	60 US medical schools offer medical Spanish experiences
Morales et al. [23]	To evaluate the state of Spanish curricula at US medical schools	Survey of 132 medical school deans	Existence of medical Spanish coursework	73 US schools have a medical Spanish curriculum
Ortega et al. [24]	To assess US medical schools’ Spanish educational efforts	Survey of 155 medical schools from March to November 2019	Medical Spanish courses offered	98/125 report some form of Spanish program; 53/98 report formal curricula

Current Trend

In 2005, 60 of 125 programs offered medical Spanish experiences, with only 26 Spanish electives apparent [22]. According to a survey done by Morales et al. in 2015, this number increased to 73 different schools implementing a formal Spanish curriculum [23]. Moreover, a recent survey conducted on this data found that 78% of schools offer some degree of medical Spanish exposure, whether it be from institutional electives, student groups, or international immersion experiences [24]. However, only 54% of current programs reported a formal curriculum [24]. Although this demonstrates an upward trend from the initial 2005 study, the number of programs providing formal instruction in the Spanish language has remained relatively stagnant over the past decade.

Five studies assessed the effectiveness of medical Spanish courses as per fluency, auditory comprehension, and student comfort [17-21] (Table 2). One study conducted was a survey among bilingual medical students assessing their level of comfort, unrelated to a medical Spanish curriculum [25] (Table 2).

**Table 2:** Summary of original studies on medical Spanish curricula and student comfort OSCE: objective structured clinical examination; SP: standardized patient; HPI: history of present illness; H&P: history and physical examination

Author	Objective or Question	Population and Design	Measured Outcomes	Main Findings
Reuland et al. [17]	Are longitudinal Spanish programs effective during medical school training?	Students (n = 45) from two cohorts at one medical school were enrolled in a four-year medical Spanish program	Speaking proficiency assessment	Baseline score: 8.7; score after two years: 9.0 (p = 0.15)
			Listening comprehension assessment	Baseline score: 77%; score after two years: 86% (p = 0.003)
Reuland et al. [18]	To evaluate the impact of Spanish immersion experience on verbal fluency with domestic coursework versus domestic coursework alone	Students (n = 56) participated in a four- to six-week medical Spanish elective in Mexico, Nicaragua, Honduras, or Peru	Increased fluency score at second-year assessment	Control: 21/46; experimental group: 45/56 (p < 0.001)
			Increased fluency score at fourth-year assessment	Control: 7/25; experimental group: 13/20 (p = 0.013)
Ortega et al. [19]	To examine change in the Spanish fluency level, comfort level, and utility of OSCEs	Students (n = 58) enrolled in a 10-week Spanish elective at one US medical school	SP- and faculty-rated fluency scores	SP-interview score: 81%; faculty-interview score: 64% (p < 0.001)
			Comfort level gathering an HPI	Pre-course comfort: ~50%; post-course comfort: >80% (p < 0.05)
Sadanand et al. [20]	To develop a standardized Spanish elective for medical schools to promote fluency	Students (n = 20) enrolled in an eight-week medical Spanish curriculum at one US medical school	Ability to obtain a medical history	Pre-elective score: 1.60; post-elective score: 3.38 (p < 0.001)
			Confidence level	Pre-elective score: ~1.9; post-elective score: ~3.3 (p < 0.001)
Vega et al. [21]	What effect do medical Spanish electives have on student comfort in Spanish-speaking interactions?	Students (n = 31) completed a 10-week Spanish course at one US medical school.	Comfort obtaining an H&P	Pre-course level: 1.29; post-course level: 3.20 (p = 0.00001)
Vela et al. [25]	To investigate medical student comfort working as ad hoc interpreters	Survey of bilingual students (n = 87) at two US medical schools (note: 41/87 were fluent in Spanish.)	Level of comfort interpreting	Uncomfortable “often” or “very often”: 11.5%; uncomfortable “occasionally”: 31%; Incident of feeling uncomfortable: 53%

Speaking Proficiency

Three different studies examined the relationship between taking a medical Spanish elective and verbal fluency [17,19,20]. In the study conducted by Reuland et al., quantitative findings showed no significant difference between speaking proficiency skills at baseline versus two-year post-assessment [17]. However, it must be taken into account that this study only recruited Spanish-speaking individuals of intermediate-advanced level. Study results by Ortega et al. and Sadanand et al. revealed a different picture [19,20]. These two studies, which included patients with low-intermediate Spanish communication skills, demonstrated a positive correlation between medical Spanish training and verbal fluency in a statistically significant manner. Hence, there is more evidence backing the effectiveness of implementing such a course. Moreover, in a 2012 study evaluating the impact of international immersion experiences on Spanish speaking proficiencies, fluency scores increased by 34% (p < 0.001) [18]. Therefore, it seems that fully immersive clinical rotations may be an effective strategy to learn a new language in a time-effective manner.

Auditory Comprehension

Only one study directly assessed listening comprehension skills. As the data exhibits, mean interim listening comprehension scores increased by nearly 10% [17]. Likewise, the proportion of individuals who successfully passed the auditory component of the examination increased from 72% to 92% [17]. Of importance, it must be considered that these results may have confounding bias given that the listening comprehension test was exactly the same at baseline versus two-year assessment.

Student Comfort

Three studies utilized a comfort scale as an outcome measure to examine Spanish curriculum efficacy [19-21]. All three studies unanimously agreed that confidence levels were significantly enhanced for students receiving formalized training in Spanish dialogue. Ortega et al. used a statistical t-test to show that 73% of students were comfortable performing simple, problem-based Spanish encounters at the one-year follow-up, with almost 90% reporting that the medical elective was useful during their first year of residency [19]. In the study by Sadanand et al., improvements were seen across all domains with respect to performing the history and physical examination, with a mean increase of 1.39 (p < 0.001) [20]. Additionally, Vega et al. demonstrated that beginner-level Spanish speakers had a remarkable trajectory, with a pre-course mean score increase from 0.53 to 3.4 (p = 0.001) [21]. Moreover, although the study conducted by Vela et al. was not related to medical school curricula, it gave us insights into the proportion of bilingual individuals feeling uncomfortable acting as ad hoc interpreters [25]. Of the 87 students included, nearly 87% were asked to act as an intermediary between the provider and patient. However, more than half of these students reported feeling uncomfortable at some point in time during a medical encounter.

Study Limitations

Nearly all of the studies were single-site reports with relatively small sample sizes. Therefore, this may lead to issues with generalizability of the results. Furthermore, several studies used non-validated self-reported surveys for data acquisition of self-assessed fluency. Also, some of the studies experienced student dropouts and others had inclusion criteria that may have skewed the results.

Discussion

In the hospital setting, numerous methods are used to communicate with Spanish-speaking Latinos with limited English proficiencies, the most common of which includes the use of interpretation services [26,27]. This has major advantages in providing care to patients, especially for physicians who lack the ability to effectively communicate in Spanish. However, one of the limitations of using a translation service includes clinically significant errors being made in dialogue propagation [27]. As most interpreters lack a structured training in medical education, they may be unable to understand the situational context, medical jargon, and the patient’s underlying history [27,28]. This can lead to words being improperly communicated in the doctor-patient-interpreter interaction. Furthermore, prior studies detailing language barriers and their impact on healthcare demonstrate that miscommunication between the patient and provider can lead to reduced adherence to treatment protocol, issues with informed consent, and inferior outcomes in patient health and well-being [13-16]. Thus, by teaching medical students basic medical Spanish terminology, they may be able to reduce the frequency of communicative errors and increase the overall comfort level of patients during clinical encounters.

As purported by numerous studies, a medical Spanish elective can be challenging to implement into the course curriculum as students present at differing baseline levels of listening comprehension and verbal fluency [17-21]. Moreover, there are limited studies analyzing efficacy of these courses with regard to the retention of course material and improvement in Spanish-speaking proficiency [22]. Other concerns include the cost of incorporating such a course as well as lack of faculty support at some institutions [23,24]. In addition, most studies commonly refer to the time commitment as being a major hurdle for medical school students to become interested in learning a new language at this stage in their career [17-25].

Taking into account the rigors of a medical school curriculum and the limited amount of time an elective can span, we argue that it would be best to gear a medical Spanish elective towards online learning modules, with a focus on empathetic communication in order to improve student-patient interactions in the Spanish-speaking community. Students should be assessed by way of daily/weekly assignments and objective structured clinical examinations (OSCEs). We believe that schools may be more inclined to offer such an elective, given that an online platform for a course may reduce costs, while still providing individuals with the opportunity to pursue their goals. Additionally, students may feel more comfortable learning a new language via an online platform due to the flexibility it provides them with during their medical training. Moreover, we would like to propose that electives are geared towards beginner-level Spanish speakers, given that most medical students in the United States are classified in this category, which can in turn impact population health on a larger scale.

In the fourth year of medical school, students are placed in a unique position. They are given the opportunity to tailor their course schedule to their preferences and take elective courses that interest them. One of the courses offered at approximately half of US medical schools across the country includes a medical Spanish elective [23,24]. As language barriers are a pivotal component that contribute to health inequities seen in the United States, more research needs to be done investigating affected communities and health outcomes [1-5]. However, we do see negative correlations between linguistic marginalization and patient care in ethnically diverse populations [7,9]. Thus, the need for healthcare providers to learn medical Spanish terminology is growing with the changing demographic and rapid expansion of the Hispanic American population.

## Conclusions

Having the opportunity to take a Spanish elective in a medical school can prove beneficial as per patient satisfaction within the Hispanic American community. In turn, this can lead to increased patient-provider interactions with an overall rise in the quality of care. It has been shown that positive interactions in a clinical context leads to increased compliance with treatment protocol as well as increased awareness and understanding of the underlying diagnosis. As such, more medical schools across the country should opt to create a medical Spanish elective with the goal of empowering students to increase the capacity for empathetic language strategies, and as a result, improve patient comfort.
